# Loss of SUMOylation on ATF3 Inhibits Proliferation of Prostate Cancer Cells by Modulating CCND1/2 Activity

**DOI:** 10.3390/ijms14048367

**Published:** 2013-04-16

**Authors:** Chiung-Min Wang, Wei-Hsiung Yang

**Affiliations:** Department of Biomedical Sciences, Mercer University School of Medicine, Savannah, GA 31404, USA; E-Mail: meowy200@yahoo.com

**Keywords:** ATF3, SUMOylation, prostate cancer, CCND, proliferation

## Abstract

SUMOylation plays an important role in regulating a wide range of cellular processes. Previously, we showed that ATF3, a stress response mediator, can be SUMOylated and lysine 42 is the major SUMO site. However, the significance of ATF3 SUMOylation in biological processes is still poorly understood. In the present study, we investigated the role of ATF3 SUMOylation on CCND activity and cellular proliferation in human prostate cancer cells. First, we showed that ATF3 can be SUMOylated endogenously in the overexpression system, and lysine 42 is the major SUMO site. Unlike normal prostate tissue and androgen-responsive LNCaP cancer cells, androgen-independent PC3 and DU145 cancer cells did not express ATF3 endogenously. Overexpression of ATF3 increased CCND1/2 expression in PC3 and DU145 cancer cells. Interestingly, we observed that SUMOylation is essential for ATF3-mediated CCND1/2 activation. Finally, we observed that SUMOylation plays a functional role in ATF3-mediated cellular proliferation in PC3 and DU145 cells. Taken together, our results demonstrate that SUMO modification of ATF3 influences CCND1/2 activity and cellular proliferation of prostate cancer PC3 and DU145 cells and explains at least in part how ATF3 functions to regulate cancer development.

## 1. Introduction

Prostate cancer is the most common cancer and one of the most prevalent causes of death in men in the USA and other Western countries. It is well known that androgens, androgen receptors, and their signaling pathways play important roles in the growth and progression of the prostate and prostate cancers. Therefore, at an early stage of prostate cancer development, inhibiting androgen receptors by antagonists and/or inhibitors is an effective therapy. However, in most patients, prostate cancer will recur due to growth and proliferation of cancer cells that are androgen-independent. Several lines of evidence suggest that most androgen-independent prostate cancers continue to express the androgen receptors as well as androgen-responsive genes, despite low or the absence of circulating androgen in these patients. Therefore, targeting androgen receptors and their associated cofactors is essential for fighting prostate cancer [1–3]. However, despite great progress in our understanding of this disease regulated by androgen, there still remains the need to explore the molecular mechanisms involved in prostate tumorigenesis and progression.

ATF3, a nuclear protein which is ubiquitiously expressed in most mammalian tissues, belongs to the basic leucine zipper (bZIP) protein family of transcription factors. ATF3 can bind to the consensus ATF/cAMP consensus sequence (5′-TGACGTCA-3′) of a number of promoters to regulate its downstream target genes, such as *DDIT3 (GADD153)*, *EGR1*, *SNAI1/2*, *TP53*, and *TWIST1* genes [4–7]. ATF3 can also bind to many proteins such as E6, p53, and smad3, resulting in alterations of cellular function [8–10]. Normally ATF3 expression is maintained at low levels in quiescent cells [11]; however, its mRNA and protein levels significantly increase upon exposure of cells to stress signals, including those initiated by genotoxic agents, infections, nerve injury, tissue damage, or physiological stress [12], thereby suggesting that *ATF3* is a stress-inducible and/or adaptive response gene. Emerging evidence has linked ATF3 in immune-surveillance and innate immune responses [13], as well as metabolic homeostasis and cardiomyocyte growth [5,14,15]. Many lines of evidence have characterized *ATF3* as an oncogene in human breast and prostate cancers, as well as in Hodgkin lymphomas [7]. Moreover, it has been demonstrated that *ATF3* is an androgen-regulated gene, and anti-androgen treatment decreases ATF3 expression in androgen-sensitive prostate cancer cells (LNCaP cells), whereas forced ATF3 overexpression stimulates androgen-insensitive prostate cancer cell (DU145 cells) proliferation [16]. However, some evidence suggests that ATF3 may be able to inhibit the process of initiating and promoting the development of tumors [17]. ATF3 expression is decreased in human colorectal cancer [18], and ATF3 overexpression results in apoptosis of human LNCaP prostate cancer cells [17]. More recently, a study showed that ATF3 represses androgen signaling by binding the androgen receptor, suggesting that ATF3 is a novel repressor of androgen signaling that can inhibit androgen receptor functions [19]. Overall, the results suggest that ATF3 plays several functional roles in cancer development and immune regulation, but the underlying mechanism remains largely unknown.

Protein modifications by the small ubiquitin-related modifiers (SUMOs) have been shown to influence and regulate a wide range of normal cellular determination processes and pathways [20–28], as well as cancer development and metastasis [29–32]. Even though the 3D-structure and conjugation pathway of SUMO are very similar to those of ubiquitin, the biological functions of SUMOylation are much different from those of ubiquitination [33,34]. SUMOylation of certain proteins prevents their ubiquitin-mediated proteasomal degradation [35,36]. SUMO modifications of the majority of the target proteins are associated with transcription regulation [28,37–41]. Extensive studies have suggested that dysregulation of SUMOylation has been associated with human diseases including cancers, developmental defects, and neurodegenerative disorders. Therefore, regulation of SUMO modification is essential and of importance for various biological processes. Previously, we demonstrated that ATF3 can be SUMOylated and lysine 42 is the major SUMO site for ATF3 [42]. However, the significance of ATF3 SUMOylation in biological processes is still poorly understood. Here we sought to explore the potential role of ATF3 SUMOylation on regulating the cellular proliferation of human prostate cancer cells.

## 2. Results

### 2.1. ATF3 Can Be SUMOylated Endogenously in the Overexpression System

**Figure 1:**
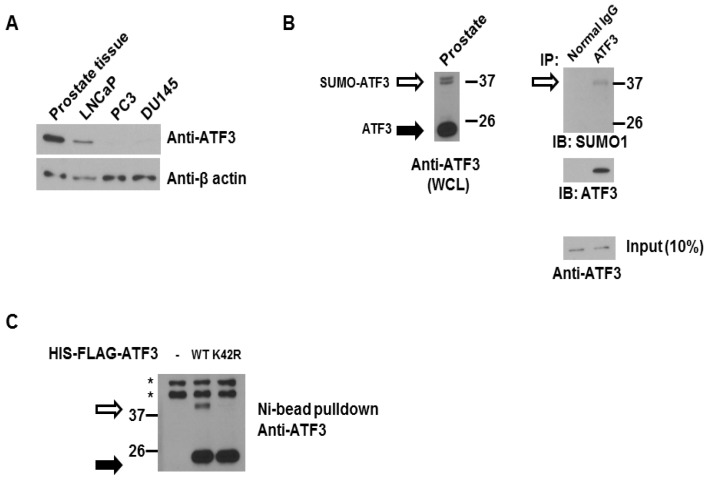
ATF3 can be SUMOylated endogenously and in overexpression system. (**A**) Total lysates from human prostate tissues and prostate cancer cells (LNCaP, PC3, and DU145) were immunoblotted with anti-ATF3 antibody. Cell lysates were probed with a β-actin antibody to control for equal loading; (**B**) Total lysate of human prostate tissue was immunoblotted with anti-ATF3 antibody (left panel). The empty arrows indicate lower mobility bands consistent with SUMOylated ATF3. The solid arrows indicate non-SUMOylated ATF3. Total lysate of human prostate tissue was first immunoprecipitated by anti-ATF3 antibody and then immunoblotted by either anti-SUMO1 or anti-ATF3 (right panel); (**C**) ATF3 SUMOylation *in vivo* in PC3 cells. Lysates of PC3 cells transfected with 3 μg HIS-tagged WT or K42R ATF3 were subjected to Ni^2+^ bead pulldown, followed by anti-ATF3 immunoblotting. The empty arrows indicate SUMOylated ATF3 and the solid arrows indicate non-SUMOylated ATF3. * indicates non-specific band.

To determine the potential role of ATF3 SUMOylation on regulating the cellular proliferation of human prostate cancer cells, we first analyzed ATF3 expression in normal prostate tissues and prostate cancer cell lines. As shown in Figure 1A, ATF3 protein levels were determined by immunoblot analysis, which showed that high levels of ATF3 were observed in normal human prostate tissue and LNCaP, androgen-dependent, prostate cancer cells. However, no or weak levels of ATF3 were expressed in PC3 and DU145, two androgen-independent cancer cells. These results demonstrate that ATF3 expression is up-regulated in normal prostate tissue and early-stage prostate cancer cells.

Previously, we demonstrated that ATF3 can be SUMOylated *in vitro* and in the mammalian cell system, and lysine 42 (K42) is the major SUMO site [42]. To confirm the previous observation, we investigated whether ATF3 can be SUMOylated endogenously by co-immunoprecipitation assay. As shown in Figure 1B, endogenous ATF3 was present in the cell lysate (WCL) of human prostate tissue and a slowly migrating ATF3-immunoreactive band around 37 kDa (consistent with SUMOylated ATF3) was detected (Figure 1B left). We next performed a co-immunoprecipitation assay and observed that SUMO1 was co-immunoprecipitated with endogenous ATF3 (Figure 1B right). We next confirmed lysine 42 of ATF3 is modified by endogenous SUMO machinery in PC3 cells. HIS-FLAG-tagged WT *ATF3* or K42R *ATF3* plasmid was transfected in PC3 cells for 48 h. The cell lysate was then precipitated by Ni-bead pulldown. As shown in Figure 1C, a SUMOylated ATF3 band was observed in cells expressing WT ATF3 but not in cells expressing K42R ATF3. From the results of the co-immunoprecipitation assay and *in vivo* SUMOylation assay, we can conclude that ATF3 can be SUMOylated endogenously and K42 is indeed the major SUMO site for ATF3.

### 2.2. De-SUMOylation Reduces ATF3-Mediated CCND1 Activation in PC3 and DU145 Cells

**Figure 2:**
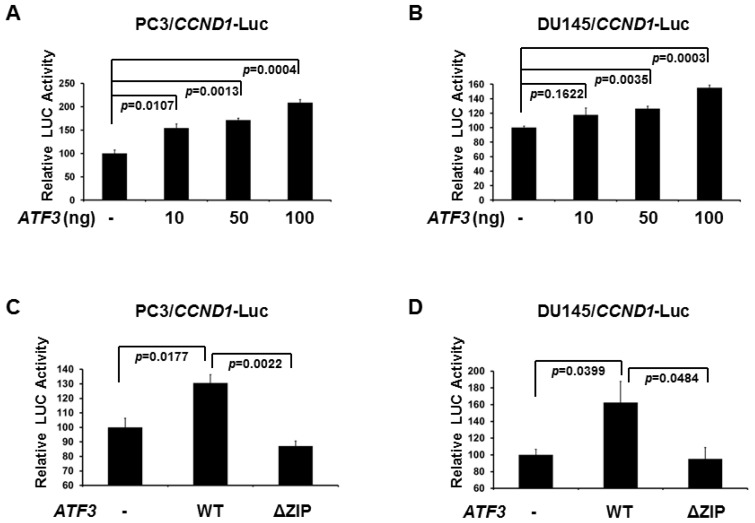
Ectopic ATF3 enhances CCND1 promoter activity. PC3 (**A**) and DU145 (**B**) cells were co-transfected with CCND1-Luc and different amount (10, 50, and 100 ng) of ATF3 plasmid. PC3 (**C**) and DU145 (**D**) Cells were co-transfected with CCND1-Luc and WT ATF3 plasmid or ΔZIP ATF3 (without DNA binding domain) plasmid. Cells were assayed for reporter activity 48 hours after transfection. Luciferase activity was measured and normalized with Renilla activity. Relative LUC activity (fold activation) was calculated and plotted. Experiments were performed three times in triplicate. Error bars indicate standard errors. *p* values were shown.

Since CCND1/2 activity is required for cell cycle G1/S transition, we decided to determine whether ATF3 influences CCND1 expression, which further regulates cellular proliferation of human prostate cancer cells. As can be seen in Figure 2A (PC3 cells) and 2B (DU145 cells), expression of WT ATF3 increased the activity of a *CCND1* promoter-driven luciferase reporter in a dose-dependent manner, while ATF3 without DNA binding motif (ΔZIP) was not able to activate *CCND1* promoter (Figure 2C,D). These data suggest that ATF3 up-regulates CCND1 expression in human prostate cancer PC3 and DU145 cells.

**Figure 3:**
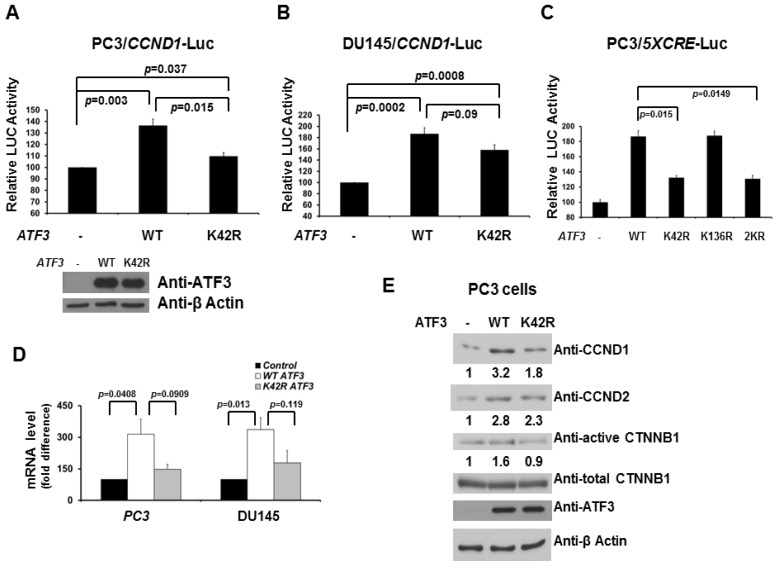
DeSUMOylation reduces ATF3-mediated CCND1/2 and CTNNB1 activity. PC3 (**A**) and DU145 (**B**) cells were co-transfected with CCND1-Luc and WT ATF3 or K42R ATF3 plasmid. Cells were assayed for reporter activity 48 h after transfection. Luciferase activity was measured and normalized with Renilla activity. Relative LUC activity (fold activation) was calculated and plotted. Experiments were performed three times in triplicate. Error bars indicate standard errors. The expression levels of WT and K42R ATF3 in PC3 cells from the reporter assays were validated using anti-ATF3 immunoblotting; (**C**) PC3 cells were co-transfected with 5XCRE-Luc and WT ATF3, K42R ATF3, K136R, or 2KR plasmid. Cells were assayed for reporter activity 48 h after transfection. Luciferase activity was measured and normalized with Renilla activity. Relative LUC activity (fold activation) was calculated and plotted. Experiments were performed three times in triplicate. Error bars indicate standard errors; (**D**) Real-time RT-PCR analysis was performed to measure CCND1 mRNA expression with GAPDH as an internal control in PC3 cells. Each point represents the average of three experiments, each with triplicate samples. Error bars indicate standard errors; (**E**) The expression levels of CCND1/2 and CTNNB1 in PC3 cells transfected with WT ATF3 or K42R ATF3 plasmid were validated by immunoblotting. Cell lysates were probed with a β-actin antibody to control for equal loading.

We next investigated the potential role of ATF3 SUMOylation on regulating CCND1 expression. Interestingly, while expression of WT ATF3 increased the activity of *CCND1* promoter-driven luciferase reporter, expression of the SUMOylation-deficient K42R mutant reduced the activity (Figure 3A,B). Similar results were observed in a 5X CRE-driven luciferase reporter assay (Figure 3C). These data also further support our previous finding that lysine 42, not lysine 136, is the major SUMO site for regulating ATF3 activity. To determine whether ATF3 alters the levels of CCND1/2 mRNA and proteins in PC3 and DU145 cells, expression vectors encoding WT *ATF3* or K42R *ATF3* were transfected into PC3 and DU145 cells. As shown in Figures 3D and 3E, when WT *ATF3* was transfected, the levels of CCND1/2 mRNA and proteins were increased three to four-fold. When K42R *ATF3* was transfected, less CCND1/2 mRNA and proteins were increased (~2 folds). Since CTNNB1 is a key downstream mediator in the Wnt signaling pathway, and an increase in active CTNNB1 has been linked to tumor proliferation and metastasis, we next investigated the role of SUMOylation on ATF3-mediated CTNNB1 expression. As shown in Figure 3E, when WT *ATF3* was transfected, the levels of active CTNNB1 proteins were slightly increased. When K42R *ATF3* was transfected, the levels of active CTNNB1 protein were unchanged. Overall, these findings link ATF3-mediated CCND1/2 activation to its SUMOylation and thereby imply this modification plays a functional role in CCND1/2 activation by ATF3 in prostate cancer PC3 and DU145 cells.

### 2.3. Loss of SUMOylation on ATF3 Reduces Proliferation of Prostate Cancer Cells

**Figure 4:**
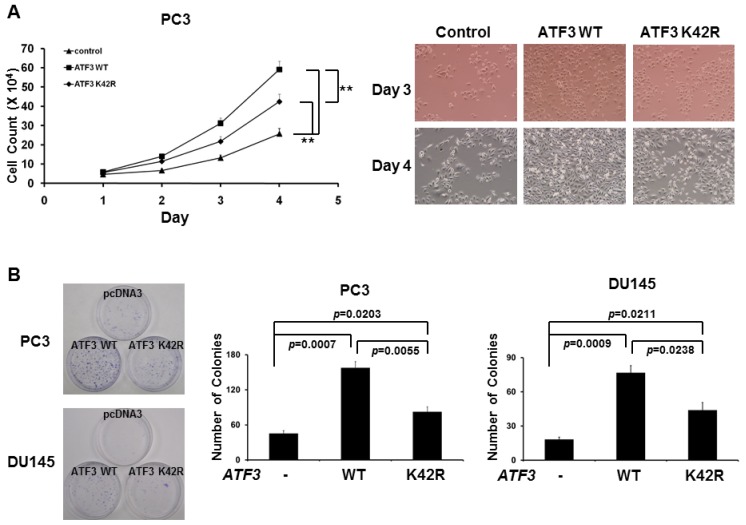
Removal of SUMOylation reduces ATF3-mediated cellular proliferation and colony formation in PC3 cells. (**A**) Cell numbers were determined in indicated time after plating of WT ATF3-expressed or K42R ATF3-expressed cells by cell counting assay. On days 3 and 4, cells were also photographed. For each test, *p* values of <0.05 and <0.001 were considered significant (*) and very significant (**), respectively; (**B**) DeSUMOylation reduced ATF-enhanced the colony formation of PC3 and DU145 cells, as shown by representative dishes of colony formation. All experiments were repeated at least three times with the same results. The graph shows the mean results and S.D. of three independent experiments.

Because SUMOylation is involved in ATF3-mediated CCND1/2 activation and CCND activity is required for cell cycle progression, we next investigated the potential role of SUMOylation of ATF3 in proliferation of prostate cancer cells. To evaluate the effect of SUMOylation of ATF3 on prostate cancer cells, recombinant pcDNA3-WT *ATF3* and pcDNA3-K42R *ATF3* were transfected into PC3 and DU145 cells and stably expressed cells were selected. The result showed that cellular growth (Figure 4A) and colony formation (Figure 4B) were significantly promoted by the enforced WT ATF3 overexpression as compared with that of those transfected with empty vector. Interestingly, removal of SUMOylation by the enforced K42R ATF3 overexpression reduced (compared to WT ATF3) cell growth and colony formation (Figure 4A,B). These collective data suggested that ATF3 overexpression plays an important role in promoting cell growth and colony formation of prostate cancer PC3 and DU145 cells by modulating CCND1/2 activity and SUMOylation is involved in and essential for this promotion.

## 3. Experimental Section

### 3.1. Reagents

All cell culture reagents and protein A-agarose were purchased from Invitrogen (Carlsbad, CA, USA). Antibodies against ATF3, CCND2 (also called Cyclin D2), and CTNNB1 were purchased from Santa Cruz Biotechnology Inc. (Santa Cruz, CA, USA). Antibodies against CCND1 (also called Cyclin D1) were purchased from Cell Signaling Technology Inc. (Danvers, MA, USA). Antibodies against β-Actin were purchased from Sigma, (St. Louis, MO, USA). Antibodies against active CTNNB1 were purchased from BD Biosciences (San Jose, CA, USA). Antibodies against SUMO1 and SUMO3 were purchased from Active motif (Carsbad, CA, USA). Luciferase activity was measured using the Dual Luciferase Assay System (Promega, Madison, WI, USA). Ni-NTA agarose was purchased from QIAGEN (Valencia, CA, USA).

### 3.2. DNA Constructs

Human ATF3 plasmid (pBP-ATF3) was kindly provided by Dr. Tsonwin Hai (Ohio State University, Columbus, OH, USA). Human WT, K42R, and ΔZIP *ATF3* pcDNA3.1(+) plasmids were previously established in our laboratory as described in Wang *et al.*[42]. The human *CCND1* promoter (−700-bp) was PCR-amplified by using forward primer 5′-ACGAGGTACCTAAAAAAAATGAGTCAG-3′ and reverse primer 5′-GCCAAGCTTCCCCGCTGCAGCCTTTC-3′ and digested with *Kpn*I and *Hin*dIII and then ligated into the *Kpn*I and *Hin*dIII sites of pGL3 to create *CCND1* promoter luciferase plasmid. 5XCRE-Luc was created by using forward primer 5′-TCGTGGTACCGTGACGTCAGTGACGT CAGTGACGTCAGTGACGTCAGTGACGTCAGCGATCTAAGTAAGCTTGGCA-3′ and reverse primer 5′-TGCCAAGCTTACTTAGATCGCTGACGTCACTGACGTCACTGACGTCACTGACGTCACTGA CGTCACGGTACCACGA-3′ and digested with *Kpn*I and *Hin*dIII and then ligated into the *Kpn*I and *Hin*dIII sites of pGL3 to create 5XCRE-Luc plasmid. All constructs were verified by nucleotide sequencing.

### 3.3. Cell Culture and Transfection

DU145, LNCaP, and PC3 cells were purchased from the American Type Culture Collection. DU145 cells were maintained in Dulbecco’s modified Eagle’s medium (DMEM) in the presence of 10% fetal bovine serum and antibiotics (GIBCO/Life Technologies, Grand Island, NY, USA) in humidified air containing 5% CO_2_, at 37 °C. LNCaP cells were maintained in RPMI-1640 medium in the presence of 10% fetal bovine serum and antibiotics (GIBCO, USA) in humidified air containing 5% CO_2_, at 37 °C. PC3 cells were maintained in F-12K medium supplemented with 10% fetal bovine serum and antibiotics in humidified air containing 5% CO_2_, at 37 °C. After incubation, the cells were transfected using Fugene HD Transfection Reagent (Roche, Madison, WI, USA). Approximately 45–48 h after transfection, the cells were harvested. Luciferase activity was measured and normalized with Renilla activity. All experiments were performed in triplicate.

### 3.4. Tissues

Protein lysates from human prostate tissues were purchased from Zyagen (San Diego, CA, USA).

### 3.5. Immunoprecipitation Assay

Cells (2 × 10^6^) were seeded onto 10-cm plates. Twenty-four hours after transient transfection, cells were harvested and lysed in lysis buffer (40 mM HEPES, 120 mM sodium chloride, 10 mM sodium pyrophosphate, 10 mM sodium glycerophosphate, 1 mM EDTA, 50 mM sodium fluoride, 0.5 mM sodium orthovanadate, 1% Triton X-100) containing protease inhibitor cocktail (Sigma, St. Louis, MO, USA), followed by rotation for 1 h at 4 °C to solubilize proteins. Soluble protein was collected and immunoprecipitated with the indicated antibody overnight. Protein A agarose beads were added to protein lysates for 2 h at 4 °C. Beads were centrifuged and washed at least three times with lysis buffer. For Ni^2+^-bead pull-down assays, Ni^2+^-NTA agarose was used to precipitate HIS-tagged ATF3 from cell lysates. Proteins were eluted by boiling in 50 μL of 2× Laemmli sample buffer, resolved by 8% SDS-PAGE, and processed for immunoblotting as described below.

### 3.6. Immunoblotting

Protein lysates were allowed to rotate at 4 °C for 30 min, and protein contents of the high-speed supernatant were determined using the BCA™ Protein Assay kit assay (Pierce/Thermo Scientific, Rockford, IL, USA). Equivalent quantities of protein (20–45 μg) were resolved on polyacrylamide-SDS gels, transferred to nitrocellulose membrane (Bio-Rad), and immunoblotted with specific antibodies. Results were visualized using the Supersignal West Dura Extended Duration Substrate kit (Pierce Chemical Co., Rockford, IL, USA). Band intensity was quantified using the ImageJ program.

### 3.7. *In Vivo* SUMOylation Assays

The *in vivo* SUMOylation assay was carried out as previously described [28]. Briefly, PC3 cells (2 × 10^6^) were seeded in 10-cm plates and 24 h later were transfected with indicated HIS-FLAG-*ATF3* (WT or K42R) expression plasmids. After 48 h, cells were harvested in 700 μL lysis buffer (500 mM NaCl, 10 mM imidazole, 45 mM Na_2_HPO_4_, 5 mM Na_2_H2PO_4_, 8 M urea, pH 8.0) containing complete protease inhibitors without EDTA (1 tablet/10 mL; Roche, Madison, WI, USA) and sonicated. Lysates were cleared and incubated with 100 μL of 50% Ni^2+^-NTA agarose (QIAGEN, Valencia, CA, USA) at room temperature for 60 min on a rotator. The resin was washed three times in wash buffer 1 (400 mM NaCl, 10 mM imidazole, 17.6 mM Na_2_HPO_4_, 32.4 mM Na_2_H_2_PO_4_, 8 M urea, pH 6.75), washed three times in wash buffer 2 (150 mM NaCl, 10 mM imidazole, 17.6 mM Na_2_HPO_4_, 32.4 mM Na_2_H_2_PO_4_, pH 6.75). Samples were resuspended in 2× EDTA SDS-PAGE sample buffer. Samples (20 μL) were resolved by 8% SDS-PAGE and processed for immunoblotting using anti-ATF3 primary antibody. Images were captured in a Kodak Image Station 440 CF using Super Signal West Femto substrates (Thermo scientific/Pierce, Rockford, IL, USA).

### 3.8. Colony Formation Assay

DU145 and PC3 cells (1000 viable cells) stably expressed WT or K42R ATF3 were seeded in 100-mm tissue culture plates with complete growth media for 11 days. Cells were fixed with methanol and stained with 0.1% crystal violet. Colonies were counted and plates were photographed.

### 3.9. Cell Proliferation Assay

PC3 cells stably expressed WT or K42R ATF3 were seeded in a six-well plate at a concentration of 5 × 10^3^ per well. At 0, 1, 2, 3 and 4 days in culture, cells were then photographed and cell proliferation was measured by trypan blue exclusion using a microscope.

### 3.10. RT-PCR and Real-Time PCR

Total RNA from PC3 and DU145 cells stably expressed WT or K42R ATF3 were extracted using TRIzol^®^ reagent and treated with DNase (Ambion/Life Technologies, Grand Island, NY, USA) to remove any residual genomic DNA, and was quantified by UV spectrometry. One microgram of total RNA was used to synthesize cDNA using the iScript™ kit (Bio-Rad, Hercules, CA, USA) according to the manufacturer’s recommended protocol. The final cDNA product was purified and eluted in 50 μL of Tris-EDTA buffer using PCR purification columns (QIAGEN, Valencia, CA, USA). Two primers (5′-CTTCGTTGCCCTCTGTGCC-3′ and 5′-CGGCCTTGGGGTCCATGTTC-3′) were used to amplify human *CCND1* fragments. Two primers (5′-CTGTGGGCAAGGTCATCCC-3′ and 5′-GGCAATGCCAGCCCCAGCGT-3′) were used to amplify human glyceraldehydes-3-phosphate dehydrogenase (*GAPDH*) fragments. For quantitative real-time PCR analysis of mRNA transcript abundance, cDNA was combined with 2× SYBR green PCR master mix (Applied Biosystems, Foster City, CA, USA), and gene-specific primers in the ABI 7,500 thermocycler system (Applied Biosystems/Life Technologies, Grand Island, NY, USA). All data were normalized to *GAPDH* as an internal standard.

### 3.11. Statistical Analysis

Statistical analyses were performed using the Student’s *t* test or a one-way ANOVA when more than two groups were compared. After the ANOVA analysis, the *post hoc* multiple comparisons were performed by using Tukey’s honestly significant difference (HSD) test to determine the statistical difference from each other among subgroups. For each test, *p* values of <0.05 and <0.001 were considered significant and very significant, respectively.

## 4. Discussion and Conclusions

ATF3 responds to a wide variety of physiological stimuli and thus functions as an important regulator of cancer development, host defense, metabolism, and cellular proliferation. Previously, we reported that ATF3 can be SUMOylated *in vitro* and *in vivo*, and lysine 42 is the major SUMO site for ATF3 [42]. Herein, we demonstrate that ATF3 overexpression increases proliferation of prostate cancer PC3 and DU145 cells and SUMOylation is involved in this enhancement by modulating CCND1/2 activities.

Normally, ATF3 levels are maintained at low levels in quiescent cells and dramatically increased upon physiological and stress stimuli. Our current results, as well as previous reports [16,43], suggest that high levels of ATF3 were observed in normal human prostate tissue and LNCaP, androgen-dependent prostate cancer cells, but no or at least weak levels of ATF3 were expressed in PC3 and DU145, two androgen-independent cancer cells, thereby suggesting ATF3 expression could be androgen regulated. Indeed, this conclusion is consistent with the previous report that ATF3 is regulated by androgen agonists and antagonists [16]. A recent report further demonstrated that ATF3 is a novel repressor of androgen signaling that can inhibit androgen receptor functions [19], suggesting that ATF3–androgen receptor interaction is well regulated in androgen-dependent prostate cancer cells. However, this interaction and regulation might be lost in androgen-independent prostate cancer cells, PC3 and DU145 cells. Therefore, targeting ATF3 would be a suitable therapeutic treatment for prostate cancer progression.

In this study, we chose PC3 and DU145 cells for ATF3 SUMOylation study mainly because (1) no or low levels of ATF3 were detectable in these two cell lines and (2) a previous report [16] showed that ATF3 stimulates DU145 cell proliferation. Consistent with the previous report, we observed that ATF3 indeed increases PC3 and DU145 cell proliferation. In addition, we further provided evidence that CCND1/2 activity is involved in ATF3-driven cell proliferation. Previously, ATF3 has shown to up-regulate CCND1 in hepatocytes [44]; however, ATF3 has demonstrated to down-regulate CCND1 in chondrocyte differentiation [45], suggesting that CCND1 activity regulated by ATF3 is tissue dependent.

In the current study, we showed that loss of SUMOylation on ATF3 reduces its ability to fully activate CCND1/2 activity, resulting in decreased cellular proliferation in PC3 and DU145 cells. This result suggests that SUMO modification on lysine 42 of ATF3 is required, at least in part, to recruit transcription co-activator(s) to fully activate *CCND1/2* gene. It is also important to address that lysine 42 is located outside of leucine-zipper domain of ATF3, which is required for ATF3–DNA binding, suggesting that SUMOylation on lysine 42 of ATF3 would not interfere its DNA binding activity directly. However, how SUMOylation of ATF3 alters its ability on recruiting transcription regulators is still largely unknown. Further studies indeed are required to dissect what transcription factor or complex is recruited by SUMOylated and deSUMOylated ATF3 on promoters of ATF3 target genes.

In summary, this study in addition to our previous findings has provided evidence that ATF3 can be post-translationally modified by SUMO, and that SUMO modification of ATF3 plays a functional role in activating CCND1/2 activity in androgen-insensitive prostate cancer cell proliferation. Because ATF3 has been demonstrated to be modified by ubiquitination [9,46] and SUMOylation [42], more studies are indeed needed to determine the interplay between SUMOylation and ubiquitination on ATF3 activity. Other post-translational modifications such as acetylation, methylation, and phosphorylation might also be involved in ATF3 function and regulation. Together, our studies add a new piece of important information to previous understanding of how ATF3 functions to regulate fundamental biological processes in the response to physiological stress and stimuli.

## Abbreviations

ATF3: cyclic AMP-dependent transcription factor-3/activating transcription factor-3
p53: cellular tumor antigen p53
TP53: gene name for p53 protein
SUMO: small ubiquitin-like modifier
SENP: sentrin-specific protease
CCND1: G1/S-specific cyclin D1
CCND2: G1/S-specific cyclin D2
CTNNB1: β-catenin
